# Investigation of coordination and order in transcription regulation of innate and adaptive immunity genes in type 1 diabetes

**DOI:** 10.1186/s12920-017-0243-8

**Published:** 2017-01-31

**Authors:** Shouguo Gao, Nathaniel Wolanyk, Ye Chen, Shuang Jia, Martin J. Hessner, Xujing Wang

**Affiliations:** 1 Systems Biology Center, National Heart, Lung and Blood Institute, NIH, Bethesda, MD 20892 USA; 2 Department of Pediatrics at the Medical College of Wisconsin and the Children’s Research Institute of the Children’s Hospital of Wisconsin, The Max McGee National Research Center for Juvenile Diabetes, 8701 Watertown Plank Road, Milwaukee, WI 53226 USA; 3 The Human and Molecular Genetics Center, The Medical College of Wisconsin, 8701 Watertown Plank Road, Milwaukee, WI 53226 USA; 4 The Department of Physics, University of Alabama at Birmingham, 1300 University Blvd, Birmingham, AL 35294-1170 USA

**Keywords:** Co-expression network, Network structure, Transcription regulation, Entropy, Type 1 diabetes

## Abstract

**Background:**

Type 1 diabetes (T1D) is an autoimmune disease and extensive evidence has indicated a critical role of both the innate and the adaptive arms of immune system in disease development. To date most clinical trials of immunomodulation therapies failed to show efficacy. A number of gene expression studies of T1D have been carried out. However, a systems analysis of the expression variations of the innate and adaptive immunity gene sets, or their co-expression network structures in cohorts at different disease states or of different disease risks, is not available till now.

**Methods:**

We utilized data from a large gene expression study that included transcription profiles of control peripheral blood mononuclear cells (PBMC) exposed to plasma of 148 human subjects from four cohorts that included unrelated healthy controls (uHC), recent onset T1D patients (RO-T1D), and healthy siblings of probands that possess high (HRS, High Risk Sibling) or low (LRS, Low Risk Sibling) risk HLA haplotypes. Both weighted and non-weighted co-expression networks were constructed in each cohort separately, and edge weight distribution and the activation of known protein complexes were examined. The co-expression networks of the innate and adaptive immunity genes were further examined in more detail through a number of network measures that included network density, Shannon entropy, h-index, and the scaling exponent γ of degree distribution. Pathway analysis was carried out using CoGA, a tool for detecting significant network structural changes of a gene set.

**Results:**

Weighted network edge distribution revealed a globally weakened co-expression network induced by the RO-T1D cohort as compared to that by the uHC, suggesting a broad spectrum loss of transcriptional coordination. The two healthy T1D family cohorts (HRS and LRS) induced more active but heterogeneous transcription coordination globally, and among both the innate and the adaptive immunity genes, than the uHC. This finding is consistent with our previous report of these cohorts sharing a heightened innate inflammatory state. The spike-in of IL-1RA to RO-T1D sera improved co-expression network strength of both the innate and the adaptive immunity genes, and enabled a global order recovery in transcription regulation that resulted in significantly increased number of activated protein complexes. Many of the top pathways that showed significant difference in co-expression network structures and order between RO-T1D and uHC have strong links to T1D.

**Conclusions:**

Network level analysis of the innate and adaptive immunity genes, and the whole genome, revealed striking cohort-dependent differences in co-expression network structural measures, suggesting their potential in cohort classification and disease-relevant pathway identification. The results demonstrated the advantages of systems analysis in defining molecular signatures as well as in predicting targets in future research.

**Electronic supplementary material:**

The online version of this article (doi:10.1186/s12920-017-0243-8) contains supplementary material, which is available to authorized users.

## Background

High throughput technologies that allow comprehensive molecular profiling at multiple levels, including the microarrays and next generation sequencing, are now in routine research use. One major challenge in systems biology is to quantitatively define the molecular states from such profiles and link them to the physiological or pathological conditions under study, and hence to identify the most relevant pathways and gene sets to complex traits, and to dissect underlying genetic architecture of the latter. Along this direction, in initial studies, sets of differentially expressed genes were used to define the molecular “signature” of a system. Genes and proteins work together in pathways or functional modules, and physiological and pathological perturbations have been shown to act through these functional units. A number of approaches have therefore been proposed to rank pathways or responsive network modules relevant to phenotype variations, including gene set enrichment analysis [1–3]. At first, most pathway analysis approaches treated genes as being independent, ignoring their interaction relationships. The molecular state of a pathway was often defined by taking the aggregate (or mean) of individual gene scores [4]. More recently, a number of new metrics have been proposed to either bias the contribution from individual genes with their topological positions in the network [1, 2], or to incorporate directly in the definition network structural measures [5–9].

In this study, utilizing a large human transcription expression data set of Type 1 Diabetes (T1D), we investigate network measures that may capture the variations of a gene set and its associations to disease. T1D is an autoimmune disease that results from the immune destruction of the insulin producing pancreatic islet β-cells. It is one of the leading chronic diseases in children. As in the study of many other complex diseases including cancer [10], a number of efforts have been made to develop gene expression based molecular signatures of disease, including those from us [11–13]. The goal is to identify biomarkers that can sensitively detect and differentiate disease processes, shed light on disease mechanisms, and hence guide the development of new intervention protocols and therapies. In a disease like T1D, where the affected human tissue (pancreatic islets) are not readily accessible (unlike cancer), peripheral blood remains the most accessible resource and represents a practical, minimally invasive surrogate for biopsy material [14–16]. However, the complex milieu of disease mediators, mainly cytokines, chemokines and other immune modulators, is often too diluted in periphery for direct detection. We have developed an alternative approach by profiling the transcription responses of control peripheral blood mononuclear cells (PBMC) provoked by these mediators [11, 13, 17–19]. Both the direct and the indirect gene expression profiling studies repeatedly indicated an innate proinflammatory transcriptional signature in T1D [11, 13–16].

The immune system consists of two arms: innate and adaptive. Together they identify and eliminate foreign pathogens, with the innate immune response responsible for the first line defense and protection from invading foreign agents, initiating and regulating the subsequent adaptive responses, which in turn is critical to control innate inflammation. In an autoimmune disease like T1D, overactive immune responses fail to differentiate the self from the non-self, and disease progression requires cell types from both the innate and adaptive immune systems. Indeed growing evidence suggests that dysregulated innate and adaptive immune responses contribute to autoimmune disorders including T1D [20, 21]. Studies in both animal models and humans have suggested that activation of innate immunity genes in the islets, innate immune response signaling pathways, induction of inflammatory cytokines and activation of adaptive immune responses, play a direct role in the pathogenesis of T1D [22–25]. For instance, T1D patients and their family members produce more IFNα in response to Toll-like receptor-9 stimulation than uHC, despite having fewer peripheral dendritic cells [26]. Gene expression studies of T1D, including both those that directly study patients’s blood cells and those that study the induced transcrption profiles in surrogates, have poininted to the inovlvedment of a number of inflammation and regulatory genes [12–16, 27]. In our previous studies we consistently observed that diabetics and their unaffected family members possess a heightened baseline innate inflammatory state centered on the interleukin 1 (IL-1) signaling [12, 13]. Despite of these findings, a systems analysis of innate and adaptive immunity genes is still not available, and how the disease is triggered and progresses remains incompletely understood.

Among the proinflammatory cytokines, IL-1, long known to directly cause β-cell dysfunction and death, has been the target for T1D therapy in several major clinical trials including the Anti-Interleukin-1 in Diabetes Action (AIDA) and TrialNet Canakinumab (TN-14) trials [28, 29]. Strong preclinical evidence supporting the design of these trials included the elevated circulating levels of IL-1 being an effective biomarker of T1D disease course, and the reduced incidence of T1D in animal models upon inhibition of IL-1 [29]. Unfortunately, like most other clinical trials for T1D and for complex diseases in general, blockade of IL-1 did not show efficacy in T1D despite the strong preclinical evidence [28, 30]. While there are many factors that could have confounded the outcomes of IL-1 antagonism in these trials and should be controlled, such as the age-dependent heterogeneity in disease course, timing of drug delivery, etc. [28, 31]; it is generally agreed that a more systems approach to immunomodulation is needed and future clinical trials should focus more on combination therapy development [28, 30–32]. A more holistic view is needed of the cell types and soluble factors involved in immune dysregulation that lead to β-cell loss in T1D. Using global transcriptional analysis, in a recent report we showed that correct immunomodulation by IL-1 antagonist therapy is evident in the AIDA and TN-14 subjects, although the therapy did not produce obvious clinical benefit [33].

In this report, using the gene expression data from our previous studies [11, 13], we investigate the till now unexplored co-expression network structure and protein complex activation in three T1D family cohorts and compared them to the uHC. We will carry out both global investigations as well a focused and systematic investigation of the adaptive and innate immunity gene sets. New disease-relevant pathway analysis will be performed using the CoGA tool [34], which emphasizes on the variations in structural orders of pathway networks.

## Methods

### Gene expression data

Human T1D gene expression data came from our previous studies [11–13], which is accessible through GEO with accession number GSE52724 and GSE35725 (http://www.ncbi.nlm.nih.gov/geo/query/acc.cgi?acc=GSE52724, http://www.ncbi.nlm.nih.gov/geo/query/acc.cgi?acc=GSE35725). Details of cohort recruitment, demographics, and experimental protocols were published previously [11, 13]. Taken these two data sets together, we further restrict the age at the time of study to be between 6 and 20 years old. The data contains transcription profiles of cryopreserved PBMC responding to the sera from 148 human subjects belonging to one of the following cohorts:

1. 44 unrelated Healthy Controls (uHC) with no familial history of any autoimmune/autoinflammatory disorder,
2. 46 Recent Onset T1D (RO-T1D) patients. Blood samples were collected after stabilization on exogenous insulin 2–7 months after diagnosis,
3. 28 autoantibody negative siblings of T1D patients that are of high genetic risk (with DR3/4 haplotypes of HLA) for T1D (HRS), and
4. 31 autoantibody negative siblings of T1D patients of low genetic risk (with non-DR3/4 HLA genotypes) for T1D (LRS).

All subjects were Caucasian, with no two subjects from a same family. All cohorts were well matched for age and gender [12]. Additionally, the transcription profiles induced by 38 of the RO-T1D sera spiked in with the Interleukin-1 Receptor Antagonist (IL-1RA) were also obtained. We previously observed elevated IL-1A in the plasma of RO-T1D (and LRS and HRS) [11]. Both IL-1A and IL-1B bind IL-1 receptor and induce the inflammation signal. IL-1RA is a natural inhibitor of the pro-inflammatory effect of IL-1 that blocks activity of both IL-1A and IL-1B and were found able to modulate IL-1 − dependent inflammation transcription signature induced by plasma collected from RO-T1D patients [12].

In total expression measurements are available for 21,998 genes (with unique Entrez gene IDs) after quality filtering. In this study, to reduce noise in data, we kept the top 15,000 genes that exhibit the highest expression variation across all samples.

### Compilation of genes important to innate and adaptive immune responses

Genes deemed by Gene Ontology (GO, www.geneontology.org) to be involved in the following Biological Processes (BP) were collected: innate immune responses (GO0045087), and the regulation of (GO0045088), positive regulation of (GO0045089), and negative regulation of (GO0045824), innate immune responses; adaptive immune responses (GO0002250), and the regulation of (GO0002819), positive regulation of (GO0002821), and negative regulation of (GO0002820), adaptive immune responses. The complete list of genes in these eight categories, together with their between-cohort difference and statistical significance are summarized in Additional file 1: Table S1 Innate & adaptive immunity genes.

### Co-expression network analysis

Both weighted and non-weighted co-expression networks were constructed. In a weighted co-expression network in cohort *l* (*l* = 1, 2, 3, 4), nodes were the genes, and its edge weight matrix was given by

1 $$ {w}_{ij}^l= abs\left({r}_{ij}^l\right), $$

where *r*_*ij*_^*l*^ is the Pearson correlation between gene *i* and *j* in cohort *l*, and *abs* standard for “absolute value”. In a non-weighted un-directed co-expression network, its adjacency matrix *A* ^*l*^ = (*A*_*ij*_^*l*^) was defined by

2 $$ {A}_{ij}^l=\left\{\begin{array}{c}\hfill \begin{array}{l}1,\kern2.75em \mathrm{if}\  abs\left({r}_{ij}^l\right)>{r}_0\ \\ {}0,\kern2.75em \mathrm{other}\ \mathrm{wise}\end{array}\hfill \\ {}\hfill\ \hfill \end{array}\right. $$

The diagonal elements were set to 0. In this study we used the hard-thresholding approach, and chose *r*_0_ = 0.75, which is around 3 standard deviations above mean abs(*r*_*ij*_^*l*^) in all cohorts *l*, when networks were constructed using 20 randomly selected samples in each cohort (to remove sample size effect).

Density of binary networks was calculated using

3 $$ \mathrm{Network}\ \mathrm{density}=\frac{1}{\ \frac{1}{2} N\left( N-1\right)}\left({\displaystyle {\sum}_{i=2}^N{\displaystyle {\sum}_{j=1}^{i-1}{A}_{i j}}}\right)\times 100\%, $$

where *N* is the network size.

The degree of disorder (or the heterogeneity) in network edge distribution was assessed using Sole and Valverde’s formulation of Shannon entropy of complex networks [35]. For a network with adjacency matrix *A* = (*A*_*ij*_), the degree *k*_*i*_ of a node *i* is given by:

4 $$ {k}_i={\displaystyle {\sum}_{all\  j\ne i}{A}_{i j}} $$

If *P*(*k*) describes the network degree distribution, then the average degree *k* = ∑_*all k*_ *k* ⋅ *P*(*k*). It follows that the distribution of the remaining degree [36] can be calculated from

5 $$ q(k)=\frac{\left( k+1\right) P\left( k+1\right)}{k}, $$

and the Shannon entropy of this network is given by:

6 $$ H(q)=-{\displaystyle {\sum}_{k=1}^{N-1} q(k) \log \left( q(k)\right)} $$

*H*(*q*) provides an measure of the network’s heterogeneity in edge distribution.

If a network is scale free, namely, its degree distribution would depend on node degree in the form of *P*(*k*) ∝ *k* ^− *γ*^, where γ is the scaling exponent, and can be determined from the slope of a linear regression of the log-log plot of *P*(*k*) versus *k*.

To further examine the structural order of co-expression networks, we also borrowed the concept of h-index that is widely used in scientific publication citation networks [37]. The h-index of a scientist is defined to be x, if he/she has published at least x papers with x or more citations each. It is designed to capture both the productivity of a scientist, and the impact of his/her work. It has been applied to study the relative importance of biomolecules such as the emergence of pathogens [38]. A recent report found that in a number of social and manmade networks, the h-index is advantageous at capturing the spreading influence of nodes when compared with node degree or coreness [39].

### Protein complex activation analysis

Protein complex (PC) annotation were obtained from the CORUM database [40] http://mips.helmholtz-muenchen.de/genre/proj/corum/index.html). This data contained 1846 human complexes, 572 of which are involved in transcription. Activation of protein complexes were assessed through degree of co-activation of their members. First, non-weighted co-expression networks were constructed for each PC, by defining its adjacency matrix AP (standing for Adjacency-Protein complex) in cohort *l* to be:

7 $$ A{P}_{ij}^l=\left\{\begin{array}{c}\hfill \begin{array}{l}1,\kern2.75em \mathrm{if}\ {r}_{ij}^l>{r}_{bg}^l+2.5\ {\sigma}_{bg}^l\\ {}\kern0.5em 0,\kern2.75em \mathrm{other}\ \mathrm{wise}\end{array}\hfill \\ {}\hfill\ \hfill \end{array}\right. $$

Where *r*_*bg*_^*l*^, *σ*_*bg*_^*l*^ are mean and standard deviation of *r*_*ij*_^*l*^ for all proteins pairs that do not appear simultaneously in any known PC. Next, the largest connected component in the co-expression network was identified. Lastly, a PC is considered activated if the largest connected component covered at least 50% of its members. Note that our approach is different from protein complex enrichment analysis proposed by others [41], in that we emphasized on co-activation of gene pairs instead of individual genes. Also note that definition in Eq. (7) did not take absolute value of the correlation coefficient, because members in an activated PC are expected to be positively correlated [42].

### Statistical and pathway analysis

Statistical analysis of the gene expression data was carried out as previously described [11, 13]. Basic pathway analysis were carried out using Gene Set Enrichment Analysis (GSEA) [3]. Gene sets and pathways whose co-expression network showed significant structural changes between different cohorts were identified using CoGA [34]. It identifies groups of “differentially *associated*” genes between two conditions, through evaluation of the Jensen-Shannon divergence in the graph spectral entropy of the two corresponding co-expression networks [34].

## Results

### Global order decay in transcription regulation in recent onset diabetics

**Fig. 1:**
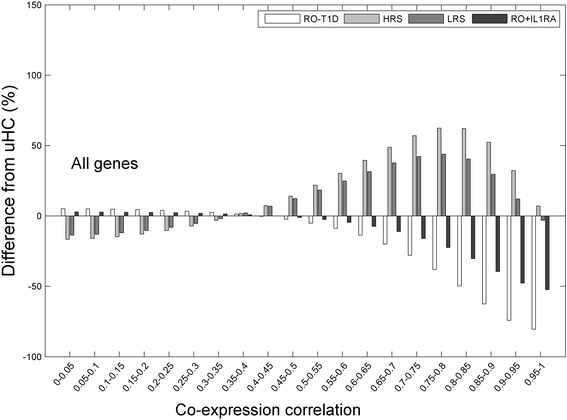
The relative difference to uHC in the distribution of co-expression network’s edge weight. The RO-T1D plasma induced much weaker co-expression networks than did plasma of the uHC cohort, with significantly less number of high weight edges. The HRS and LRS cohorts exhibited a trend opposite to that of the RO-T1D. IL-1RA spike-in to the RO-T1D sera moderately improved the co-expression strength

Co-expression networks generated from cross-sectional cohorts are good proxies to study the organizations and order in transcription regulation. We first constructed weighted co-expression networks. To eliminate potential bias brought by the different cohort sample sizes, we randomly sampled 20 subjects from each cohort 20 times, and determined the means and standard deviations of the measures. All edges were partitioned into 20 evenly distributed bins based on their mean weight (from the 20 random samplings). To examine the difference in cohorts and to highlight the deviations from what would be expected in normal individuals, we contrasted the 3 T1D family cohorts (RO-T1D, HRS, and LRS) against uHC, and the results were given in Fig. 1.

Overall there are less co-expression coordination induced by the RO-T1D sera than that by the uHC sera, with more number of weak edges and significantly less number of strong edges. The number of edges with correlation above 0.85 was over 30% less, and the number above 0.75 was ~16% less. The significance in the observed difference was evaluated through sample permutations, and was found to be *p* < 0.001. The much weakened co-expression coordination in RO-T1D indicated a broad spectrum dysregulation and lack of control in transcription. Spike-in of IL-1RA to the RO-T1D plasma restored to a moderate degree the transcription coordination among genes.

Siblings of T1D patients have an estimated 6% probability of developing diabetes, ~ 15-fold higher than that of the general population. In Caucasians the majority, >90%, of those who progress possess a high-risk DR3 and/or DR4 HLA haplotype, as compared to a carrier frequency of approximately 40% [43]. In Fig. 1, the two heathy T1D sibling cohorts (HRS and LRS) exhibited opposite trend to RO-T1D cohort, with significantly elevated global transcription coordination than the uHC.

We further examined the order of global transcription regulation from the prospective of protein complex (PC) activation. PCs are the smallest functional and structural units of the protein-protein interaction network, carrying out most of the vital and basic cellular functions. For instance, transcription regulation by transcription factors (TF) is most often carried out by binding of TF-formed protein complexes to the promoter region of genes. Over one thousand human protein complexes have been experimentally validated so far [40]. Several reports suggested that human diseases are closely associated with protein complexes [44, 45]. A functionally relevant transcription induction requires designed transcription regulations; reversely, an orderly (less chaotic or less heterogeneous) activation of transcription regulation likely would result in more coordinated activation of functional modules. Therefore we expect that examination of PC activation can offer a perspective of the order in transcription regulation.

Identification of activated protein complexes requires different algorithms from those typically used in pathway or gene set enrichment analyses. Pathways or gene sets normally contain multiple modules, with positive and negative feedback loops as well as redundancies in signaling and regulation. The interactions among members are usually sparse. We do not expect all members in a pathway to be uniformly co-activated or co-suppressed in a biological process of interest; and if a critical module or signaling path is activated, we may consider the whole pathway activated. In contrast, a protein complex is an entity where members are physically associated with each other and acts as one functional unit; its activation requires all its members to be co-activated. Therefore, we expect that members of an activated protein complex to show a high positive correlation in their expression variations (i.e., high degree of co-expression) [42]. In our dataset, we indeed found that gene pairs appearing in the same protein complexes showed higher co-expression than gene pairs that are not (Additional file 2: Figure S1).

Using the approach described in Methods, we identified activated protein complexes in each cohort, and the numbers were summarized in Table 1. We only kept those of size 5 (with maximally 10 network edges) or above, smaller protein complexes have too few subunits for meaningful co-expression assessment. The complete list of activated PCs in all cohorts and their functional annotations is available in Additional file 1: Table S2. The top three functional categories shared by them are cell cycle, metabolism and transcription. Overall the uHC cohort activated a much higher number of PCs than the three T1D family cohorts (19, versus 10, 1 and 4 for the RO-T1D, HRS, and LRS cohorts, respectively). Among the PCs activated in the three T1D family cohorts, there was also significant fewer that are involved in transcription (Table 1, in parenthesis). The results indicated a global order decay in induced transcription regulation by the three T1D family cohorts, and hence much fewer functional modules (i.e. PC) of the basic cellular biological processes were activated. Taken Fig. 1 and Table 1 together, the findings suggested that compared to the uHC, the RO-T1D induced globally weakened and more disordered coordination in transcription among genes, with fewer activated functional modules; the two healthy T1D families induced more active, but more heterogeneous/disorderly global gene-pair level coordination, such that the higher number of co-expressed gene pairs did not lead to activation of more functional modules. Interestingly, the spike-in of IL-1RA to the RO-T1D sera, though only mildly restored the global pairwise co-expression (Fig. 1), significantly increased number of activated PCs (26, even higher than the 19 of the uHC, Table 1), with a similar proportion that are involved in transcription to the uHC (11 out of 26, 42%, versus 7 out of 19, 37%). The results suggested that by spiking-in IL-1RA, we have introduced a strong regulatory signal into those co-cultures that seemingly restored order.

**Table 1:** Number of protein complexes activated in each cohort (only PCs of size 5 and above were considered)

uHC	RO-T1D	HRS	LRS	RO + IL-1RA
19 (7)^a^	10 (1)	1 (0)	4 (1)	26 (11)

^a^in parenthesis are the numbers of protein complexes involved in transcription

### Co-regulation analysis of genes involved in innate and adaptive immunity

The complete list of genes in the eight GO biological processes categories related to innate and adaptive immune responses, together with their between-cohort Fold Change (FC) and statistical significance are summarized in Additional file 1: Table S1 Innate & Adaptive Genes. We first examined reports of expression changes of their member genes in existing gene expression studies.

In our first report of patient plasma induced transcrption profiles in surrogates [13], we observed that the plasma of RO-T1D triggered a partially IL-1 dependent signature, which included induction of IL1B, CCL2, CCL7, ICAM1 and PTGS2, relative to the plasma of uHC. CCL2 and ICAM1 are known innate immune response genes and IL1B is a adaptive immune response gene, according to GO (Additional file 1: Table S1). We found that establishment of this signature preceded both the development of autoantibodies and clinical diagnosis by up to 5 years [13]. Consistent with our observations, others also reported a proinflammatory, IL-1 biased signature in cultured human islets exposed to T1D plasma [27]. In our subsequent studies, we found that relative to the uHC, an elevated, partially IL-1 dependent inflammatory state was also present in unaffected siblings of T1D probands [12, 13]. Interestingly, among the three T1D cohorts, the signature of the LRS was the most distinct from the uHC, and with the most robust induction of innate inflammatory transcripts. The genes induced included IL1B, CCL2, CCL3, CCL7, CXCL1, CXCL2, CXCL3, CD14 and TREM1, where CCL2, CCL3, CD14, and TREM1 are annotated by GO to be innate immune response genes, and IL1B an adaptive immune response gene (Additional file 1: Table S1). We also observed that this familial inflammatory state displayed greater evidence of being immunoregulated among the HRS compared with the LRS. The HRS signature exhibited more robust induction of IL-10/TGF-β dependent transcripts, suggesting more active immunoregulatory mechanisms. The genes induced included TGFBR2, SMAD9, SMAD5, SKI, SKIL SMURF1, SMURF2, FCGR2B, PIAS1, CASP8, and LGALS3, etc. Among them, PIAS1 CASP8 and LGALS3 are involved in innate immune response (Additional file 1: Table S1).

The studies that directly profiled gene expression variations in patient PBMC also reported many genes in the innate and adaptive immune responses. Kaizer et al. [14] found that PBMCs of pediatric RO-T1D exhibited an innate inflammatory transcriptional profile that included elevated IL1B, PTGS2, CXCL1, EGR2, EGR3 and TREM1 levels, which resolved in the months after diagnosis. Stechova et al. examined the transcriptional profiles of PBMCs isolated from pediatric RO-T1D, their healthy autoantibody-negative first-degree relatives and uHC, and found that the most significantly altered immune response pathway was IL-1 signaling [15]. A study by Reynier et al. [16] that investigated gene expression profiles of unfractionated whole blood reported an IFN-regulated signature that was associated with pediatric RO-T1D and their autoantibody-positive first-degree relatives. The signature included expression of IFI27, OASL, ISG15, IFIT3, GBP1, IFIT2, IFIT1, OAS3, STAT1, RSAD2, IFI44A, all but IFI44 are innate immune response genes (Additional file 1: Table S1).

In summary, a number of individual innate and adaptive immune response genes (collected in Additional file 1: Table S1) showed significant differential expression in T1D. We found that, however, conventional gene-level analysis (heatmaps, volcano plots, etc.) were not able to reveal any clear set-level pattern in the eight innate and adaptive immunity gene sets (Additional file 2: Figures S2 and S3). The enrichment for differentially expressed genes was moderate, with odds ratio ranged 1.25 to 1.37 for the eight GO BP categories, and dropped to around 1 when only genes of comparable expression levels were included (genes of known functions, such as those in these eight GO categories, tend to be expressed at higher levels than average genes). Indeed, in our previous reports, none of the eight categories came up on top in pathway analysis (see Table 3 of [13], Table 1 of [11]). Using the GSEA tool [3] we performed a focused enrichment analysis of these eight gene sets only (thus mostly avoiding the need of the multiple-pathway testing correction). Only two categories were significant between RO-T1D and uHC (Additional file 1: Table S3): innate immune responses (GO0045087), and the negative regulation of innate immune responses (GO0045824). This analysis demonstrated the utility as well as the challenges when using a conventional statistical and pathway analysis approach: while it is good at providing a first pass global picture, it may miss some biologically relevant pathways. There are several reasons for this. One is the multiple-testing correction problem when there is no prior hypothesis, as one need to test all the available pathways/gene sets. Another reason, which is often overlooked, is the fact that the expression level and the variation of a given gene is dependent on its ontology/function [46]. Genes occupying important positions in interaction networks and playing regulatory roles (e.g. those that interact with many, or influence or regulate the expression of other genes) often exhibit smaller dynamic ranges of expression variations [47, 48]. In fact, in our previous report we observed that the induction of transcripts encoding proinflammatory mediators by T1D plasma was more robust than the induction of regulatory transcripts in the signatures of healthy controls in cross-sectional analyses [12].

**Fig. 2:**
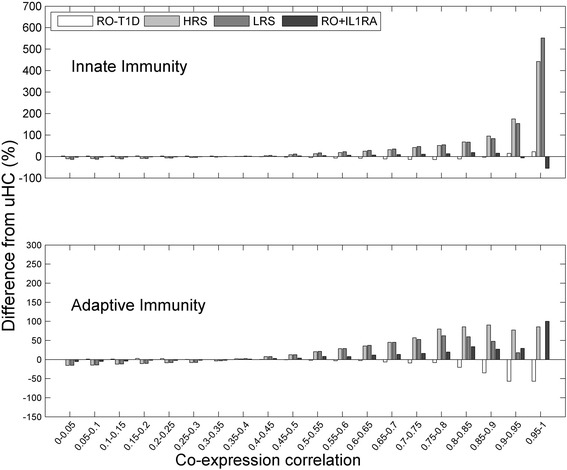
The relative difference to uHC in the distribution of co-expression network’s edge weight, for the innate and adaptive immunity genes. Genes involved in innate immunity showed stronger co-expression in all T1D family cohorts, while genes involved in adaptive immunity, exhibited weaker coordination in HRS. The LRS has no data in the last bin

While integration with biological knowledge can help to alleviate the multi-testing problem by focusing only on those pathways with prior hypothesis, incorporating consideration of interaction structure can help to address the second challenge and improve the sensitivity of detection of biologically-relevant pathways. In our dataset, when we examined the co-expression network structure of genes involved in innate and adaptive immunity, more interesting observations emerged. The edge weight distributions of the co-expression networks were presented in Fig. 2. All T1D families show striking differences in transcription coordination from uHC. Interestingly, transcription coordination of the innate and adaptive genes induced by the RO-T1D showed opposite trend from that induced by the uHC, with the adaptive genes following a similar trend to that of the background genes and innate genes following an opposite trend (compare Figs. 1 and 2).

We previously observed elevated IL-1A in the plasma of RO-T1D (and LRS and HRS) [11]. IL-1RA is a natural inhibitor of the pro-inflammatory effect of IL-1 that blocks activity of both IL-1A and IL-1B and we found it able to modulate IL-1 − dependent inflammation transcription signature provoked by the RO-T1D plasma [12]. Interestingly, the introduction of IL-1RA to the RO-T1D sera overall made the RO-T1D behave more like the two healthy T1D family cohorts (HRS and LRS).

In our data, the expression measurements were available for 842 innate and adaptive immunity genes. Only around one quarter of them are annotated in the CORUM protein complex database, too few for a proper protein complex activations analysis as we did in the previous section.

### Network structural measures of genes involved in innate and adaptive immunity

**Fig. 3:**
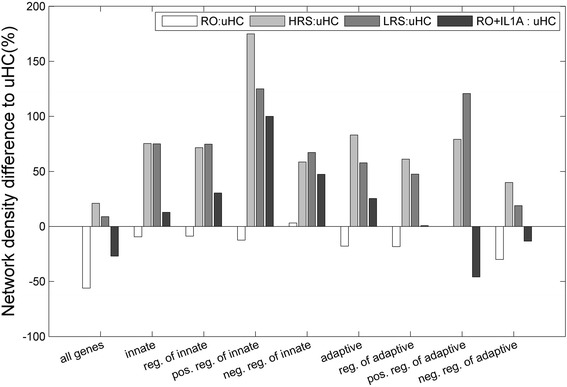
The co-expression network density of the innate and adaptive genes in each T1D family cohort as compared to the uHC cohort, showing distinct cohort difference

**Fig. 4:**
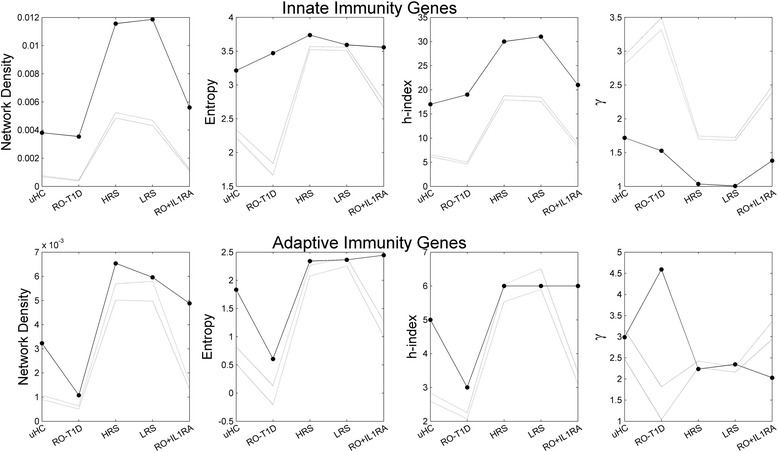
Structural measures of co-expression networks of innate and adaptive immunity genes. Dashed lines represent the 95% confidence interval of randomly selected gene sets of the same size

We further constructed unweighted co-expression networks as defined by Eq. (2) and compared the density and a set of structural measures (Figs. 3 and 4). In general the co-expression networks are sparse, with density ranging between ~0.1-1%. The relative difference to uHC of each cohort is given in Fig. 3. Again, the global transcriptome induced by RO-T1D exhibited lower network density, indicating weakened transcription coordination, while those induced by HRS and LRS showed high global coordination. The two T1D family cohorts HRS and LRS largely behaved similarly, except for genes involved in positive regulation of innate or positive regulation of adaptive immune responses, potentially responsible for their differential disease risk. These two gene categories are also the ones where their genes shows the most elevated coordination in the two healthy T1D family cohorts compared to uHC. The addition of IL-1RA reversed the trend of RO-T1D in majority, six out of eight, of the gene categories (Fig. 3).

In Fig. 4, the network measures are also compared with random selected, same number of, genes in the same cohort (dashed lines, 95% CI obtained through 500 random samplings). Note that since the innate and adaptive networks are of different sizes, it is not meaningful to directly compare them; instead, we should compare the differences to their corresponding controls. Z scores of the network measures (against random gene networks of the same size) are available in Additional file 2: Figure S4. In our previous report [12, 49], by examining the expression levels of key inflammation genes we found that the LRS signature exhibited the most robust induction of innate inflammatory transcripts; and that the HRS signature exhibited more robust induction of active immunoregulatory mechanisms, as reflected by IL-10/TGF-β dependent transcripts. The HRS, possessing high-risk HLA haplotypes, is also expected to exhibit increased likelihood of an adaptive response and diabetes progression. Interestingly, from Fig. 4, the LRS cohort induced the most dense co-expression network of the innate immunity genes while the HRS of the adaptive immunity genes. We examined the top hubs in each co-expression intranet (defined to be the top 5 ranked genes in terms of network degree in the corresponding co-expression network, Additional file 1: Table S4). There are in total 21 hubs observed in the innate intranet in the 5 cohorts, with 5 of them shared in two or more cohorts: FCER1G, TYROBP, DUSP6, OAS2, HERC5. Two of them FCER1G, and TYROBP are hubs in 3 cohorts: uHC, RO-T1D, and RO + IL-1RA; DUSP6, is a hub in both RO-T1D and LRS; and OAS2, HERC5, are hubs in both UHC and HRS. FCER1G is also a hub in RO-T1D adaptive intranet. The adaptive co-expression intranets did not share any hubs. Some of the hubs were reported previously to be active inflammation or regulatory genes (IFIT3 [16], a hub in HRS innate intranet, LGALS3 [12, 13], a hub in LRS adaptive intranet, Additional file 1: Table S4).

The background co-expression network landscape is also strongly cohort dependent. Note that as we indicated in methods, these cohorts were all from a same race (Caucasian), matched for age and gender, each of a large sample size (ranged 28–46, totaling 148 subjects). Therefore the background difference likely is related to their difference in disease status, and the genetic and environmental risks for disease. In all cohorts, the immune response genes are more coordinated with each other than background genes. In addition, the innate immunity genes formed much denser networks than background genes, as compared to the adaptive immunity genes, and exhibiting the highest density in the HRS and LRS cohorts. Whether it is the innate or the adaptive immunity genes, their co-expression networks were the weakest in the RO-T1D among all 5 cohorts.

The innate immunity genes showed a higher degree of coordination in all the T1D family cohorts than was observed in the uHC, with the most significant elevation observed in the LRS cohort. This is consistent with our observations of an elevated innate state amongst T1D family members [12]. The RO-T1D induced the lowest degree of immune gene coordination might be surprising at first. However, T1D results from the autoimmune destruction of the pancreatic islet β-cells; by the time of onset, majority of the β-cells are already destroyed, and the autoimmune activities has passed its prime.

The results of the other network measures (entropy, h-index, γ) for innate and adaptive immunity genes are also summarized in Fig. 4 (second to fourth columns, and Additional file 2: Figure S4). The Shannon entropy reflects the heterogeneity (or lack of order) in the network edges [35], i.e., in the co-expression coordination among genes. In all cohorts both the innate and adaptive genes showed higher entropy in their coordination (indicating higher heterogeneity) than random genes (Fig. 4, second column). This is likely contributed partially by their much higher network density (Fig. 4, first column). The RO-T1D induced innate immunity gene transcription exhibited the highest elevation in entropy from the random background genes. Putting the three T1D family cohorts together, the results suggest that in the two healthy cohorts the innate genes coordinate with each other significantly more than expected by chance, and the increased interaction did not lead to significant increase in entropy suggesting strong order in transcription regulation; in contract, the RO-T1D patients also induced higher than expected interaction among the innate genes, most of increase are heterogeneous and chaotic, leading to much increased entropy. The cohort-dependent entropy variation of adaptive immunity genes is similar to that of the background genes.

The functional module (i.e. protein complex) activation analysis carried out in the previous section suggested that there is global order decay in all three T1D family cohorts. At the order of magnitude level, the two healthy T1D cohorts (HRS and LRS) barely activated any protein functional modules (the numbers are in single digits, 1 & 4), in contrast to the double-digit protein complexes activated by the other three cohorts (19, 10, and 26 for uHC, RO-T1D, and RO + 1L1RA, respectively). At bulk level, this is consistent with the much higher entropy values of the background networks induced by the HRS and LRS cohorts in contrast to the other three cohorts.

The h-index suggested that there are more high-impact, influential hub gene nodes in the innate or the adaptive immunity intranet than random. The cohort dependent variations could largely be explained by the network density differences. The co-expression networks showed good scale-free behavior, both for the innate or adaptive immunity genes, as well as for the whole transcriptome (Additional file 2: Figure S4). The results of the scaling exponent γ were consistent with the other network measures. In general, and as expected [35], the lower the value of γ is, the more distributed the network is, with higher entropy, and a higher number of hub nodes (i.e. higher h-index).

In this study the co-expression network was constructed using the simple Pearson-correlation-based adjacency matrix approach. We also investigated other approaches including using the topological overlapping matrix, the findings are similar (data not shown).

### Pathways that showed significant difference in network structural order between RO-T1D and uHC

In the previous sections, we have shown that the diseases-relevant pathways/gene sets (innate and adaptive immune responses) exhibit significant changes in their network structural measures, but may not be identified by the conventional pathway or gene set enrichment analysis approaches. The results highlighted the importance of a pathway analysis approach that incorporates the consideration of network measures. Using CoGA [34], we investigated the KEGG pathways (http://www.genome.jp/kegg/pathway.html) whose co-expression network structure is different between RO-T1D and uHC. CoGA compares a variety of network structural features including the graph spectral entropy, to determine the statistical significance of network alterations [34]. Those with *p* < 0.05 are listed in Table 2. In the previous studies of this same data set, we used more conventional pathway analysis approaches that did not incorporate network structure considerations (see Table 3 of [13], and Table 1 of [11]). We can see that the CoGA was able to uncover a number of new pathways.

**Table 2:** KEGG pathways whose co-expression networks are significantly different in structure between the RO-T1D and uHC, as identified by CoGA

KEGG pathway ID	Pathway name	Pathway size	Nominal *p*-value
HSA00072	Synthesis and degradation of ketone bodies	9	0.00099
HSA04940	Type I diabetes mellitus	43	0.0040
HSA03430	mismatch repair	23	0.012
HSA00604	Glycosphingolipid biosynthesis - ganglio series	15	0.017
HSA03420	Nucleotide excision repair	46	0.017
HSA00563	Glycosylphosphatidylinositol (gpi)-anchor biosynthesis	25	0.019
HSA05416	Viral myocarditis	70	0.020
HSA04142	Lysosome	120	0.022
HSA04145	Phagosome	148	0.025
HSA05140	Leishmaniasis	73	0.027
HSA05330	Allograft rejection	37	0.030
HSA03008	Ribosome biogenesis in eukaryotes	74	0.033
HSA03030	Dna replication	36	0.038
HSA05110	Vibrio cholerae infection	54	0.042
HSA00603	Glycosphingolipid biosynthesis - globo series	14	0.049

The top pathways are clearly associated to T1D, with the number two being the pathway of “HSA04940: TYPE I DIABETES MELLITUS”. Many of the genes in this pathway (Additional file 2: Figure S5) are involved in inflammation or immune regulations (IL-1, IFγ, TNFα, MHCII, etc.). The number one significant pathway is HSA00072: SYNTHESIS AND DEGRADATION OF KETONE BODIES (Additional file 2: Figure S5). Most hospital admissions of T1D occur as a result of diabetic ketoacidosis, a condition of overly high level of ketone bodies, which is a direct consequence to a body’s lack of insulin. Ketoacidosis predominantly occurs in those with T1D [50]. The number three pathway The (DNA) Mismatch repair pathway (Additional file 2: Figure S5) has also been associated with T1D. Defects in mismatch repair results in minisatellite and microsatellite instability. The allelic variation in minisatellites has been associated with T1D risk [51, 52]. Many other pathways in Table 2 have also been linked to T1D, for instance, both mathematical modeling and laboratory studies have indicated that impaired phagocytosis contribute to T1D [53, 54]. We propose that these pathways offer new insights to the disease pathogenesis of T1D, and maybe new candidate targets for intervention.

## Discussion

As stated by Linus Pauling: “Life is a relationship among molecules and not a property of any molecule.” In complex systems, structure defines function. The genomes of higher organisms devote a larger proportion of genes in regulations, and their complexity lies in the more sophisticated network structures rather than number of genes. Complex traits and diseases result from the interactions of many genes within a dynamic environmental background. Disruption of genetic network architecture is believed to contribute to many complex diseases. In this study, our analysis revealed striking differences in the co-expression network structural measures suggesting their potential in cohort classification, molecular state definition, and disease-relevant pathway identification. While the results were encouraging, they also revealed many challenges in network modeling. There was no single network measure that performed the best at capturing the cohort-dependent difference. Recently we have seen an increase in the appreciation of the importance of network structure, and a boom of the “third generation” pathway analysis tools that incorporate the consideration of network structure [7, 9]. However, while a number of structural metrics have been proposed, there is no clear winner [55]; it is still not clear how to best characterize the functional impact of network structural variations, nor is it known whether we can depict the molecular state of a network using one or several numbers. Is it feasible to identify an “orthonormal basis” to characterize the network structure? More efforts are warranted to answer these questions.

Note that a network-based approach complement the more conventional gene-based approach, they are not to replace each other; rather, they can be and should be integragted. While a gene-based approach is good at quickly identifying genes exhibited the most significant variations, a network-based approach is able to provide a more systems picture, revealing what interactions between genes have changed. Given the complexity of human physiology and disease, and the current incomplete understanding of their genetic architecture, we are somewhat like the blind men and an elephant, using different approaches to obtain different perspectives. When integrated, they together will hopefully give us a picture that is closer to the truth. Most of the gene expression studies in T1D till now used the gene-based approach, and were able to identify the inovlvedment of a number of inflammation and immune regulatory genes [12–16, 27]. In this study, through a systems network-based approach, we showed that healthy T1D family cohorts exhibited distinct gene coordination characteristics from that of the unrelated healthy controls, particularly the more active but more chaotic coordination of innate immune response genes, likely have resulted from the common genetics and/or evironmental factors shared among the T1D family members. This is also consistent with our previous report of these cohorts possessing a heightened innate inflammation state based on gene-level observations [12, 13]. The immune system is not isolated from other components in the human body. All network structural measures also showed clear cohort-dependent differences for randomly selected genes from the whole transcriptome (Fig. 4), indicating global differences in the transcription regulation landscape. If and how this landscape difference contribute to risk for autoimmunity? Or is it a result of the underlying defects in immune regulation? Is it related the genetic disposition to T1D (non-DR3/4 HLA genotypes, etc.)? These questions worth further investigation and the answer will help us to determine the molecular and physiological state of the body, and within the context, understanding disease development.

Numerous single drug agent clinical trials for the major common complex diseases have been carried out, overwhelming majority of them failed to show benefit. In T1D none of the recent trials of IL-1 antagonism demonstrated efficacy, despite the mounting evidence from animal model and preclinical studies of an important role the IL-1 signaling pathway played. The need of developing combination therapies is now increasingly appreciated [31]. In T1D it has been purported that a successful combination therapy will need consider all major players and their interactions: agents that modulate the innate and adaptive immune systems, and agents that preserve β-cell health and function [32]. However, implementation is not easy, design of the right combination is a challenge, and the few attempts of combination therapy in T1D have to date mostly failed [32]. A systems evaluation of the transcriptomic states and the definition of quantitative measures of such states will shed light to the identification of key agents and the design of their combinations in therapy. In our previous report we showed that although the IL-1 antagonism failed to generate positive clinical outcome in AIDA and TN-14, from blinded analysis of expression profiles alone, we were able to correctly call 70.2% of AIDA and 68.9% of TN-14 subjects to their treatment arm (treated or placebo) [33]. In the current analysis, we showed that spike in of IL-1RA restored the order to a moderate degree in transcription regulation globally, affected the adaptive immunity gene network more than the innate network (Fig. 4).

## Conclusions

In this study, we found a significant, broad spectrum global weakening in the transcription regulation induced by the RO-T1D cohort plasma in surrogate PBMC, and increased heterogeneity and loss of order in that induced by all three T1D family cohorts. In addition all T1D family cohorts induced more active but heterogeneous and disorderly transcription of the innate immunity genes, consistent with our previous report of the existence of a heightened basal innate inflammatory state in them. Spike-in of IL-1RA partially alleviated the dysregulation in innate immunity genes. Whether it is co-expression or protein interaction networks, we found that the cohorts showed more striking difference in network structures and could be clearly discriminated, than in gene expression measurements alone. We further employed CoGA, a graph theory based pathway analysis tool [34], to identify pathways exhibited network structural changes between RO-T1D and uHC. A number of new pathways were predicted, which, under close examination, displayed strong disease relevance and hence potential of being noval targets for intervention. Our results demonstrated both the importance of a systems data analysis in providing insights to the genetic architecture of disease disk, as well as the challenges in such an analysis.

## Additional files

Additional file 1:
**Table S1.** Innate & adaptive immunity genes. **Table S2.** Activated protein complexes. **Table S3.** GSEA results of the expression difference of innate and adaptive immunity genes between RO-T1D and uHC. **Table S4.** Hub genes in co-expression networks of innate & adaptive immunity genes. Hub genes are fined to be those whose network degrees are in the top 5. (XLSX 484 kb)
Additional file 2:
**Figure S1.** Genes in the same protein complexes show higher co-expression than genes that are not. **Figure S2.** Expression heatmap of the adaptive and innate immune response genes. **Figure S3.** Volcano plots that compares the distribution of the innate and adaptive immune response genes (red) against all genes (black), showing no obvious deviation. **Figure S4.** Z-scores of the network measures presented in Fig. 4. Solid lines: innate network; dashed lines: adaptive network. **Figure S5.** The intranet of the innate and adaptive immunity genes exhibit good scale-free behavior. **Figure S6.** Top 3 KEGG pathways (see Table 2) that are different in co-expression network structure between RO-T1D and uHC, as identified by CoGA. Color of a node indicates the expression log_2_FC of the corresponding gene between RO-T1D and uHC. (PDF 408 kB)

## Abbreviations

HRS: High risk sibling
LRS: Low risk sibling
RO: Recent onset
uHC: Unrelated healthy control
