# Supplementary material for: Investigation of coordination and order in transcription regulation of innate and adaptive immunity genes in type 1 diabetes
Source: BMC Med Genomics. 2017 Jan 31;10:7. doi: 10.1186/s12920-017-0243-8 (PMC5282641; doi:10.1186/s12920-017-0243-8)
Supplement: Additional file 2: — Figure S1. Genes in the same protein complexes show higher co-expression than genes that are not. Figure S2. Expression heatmap of the adaptive and innate immune response genes. Figure S3. Volcano plots that compares the distribution of the innate and adaptive immune response genes (red) against all genes (black), showing no obvious deviation. Figure S4. Z-scores of the network measures presented in Fig. 4. Solid lines: innate network; dashed lines: adaptive network. Figure S5. The intranet of the innate and adaptive immunity genes exhibit good scale-free behavior. Figure S6. Top 3 KEGG pathways (see Table 2) that are different in co-expression network structure between RO-T1D and uHC, as identified by CoGA. Color of a node indicates the expression log2FC of the corresponding gene between RO-T1D and uHC. (PDF 408 kB) [file 12920_2017_243_MOESM2_ESM.pdf]

Supplementary figures

Figure S1. Genes in the same protein complexes show higher co-expression than genes that are not.

Figure S2. Expression heatmap of the adaptive and innate immune response genes.

Figure S3. Volcano plots that compares the distribution of the innate and adaptive immune response genes (red) against all genes (black), showing no obvious deviation.

Figure S4. Z-scores of the network measures presented in Figure 4. Solid lines: innate network; dashed lines: adaptive network.

Figure S5. The intranet of the innate and adaptive immunity genes exhibit good scale-free behavior.

Figure S6. Top 3 KEGG pathways (see Table 2) that are different in co-expression network structure between RO-T1D and uHC, as identified by CoGA. Color of a node indicates the expression $\log_2$FC of the corresponding gene between RO-T1D and uHC.

Figure S1

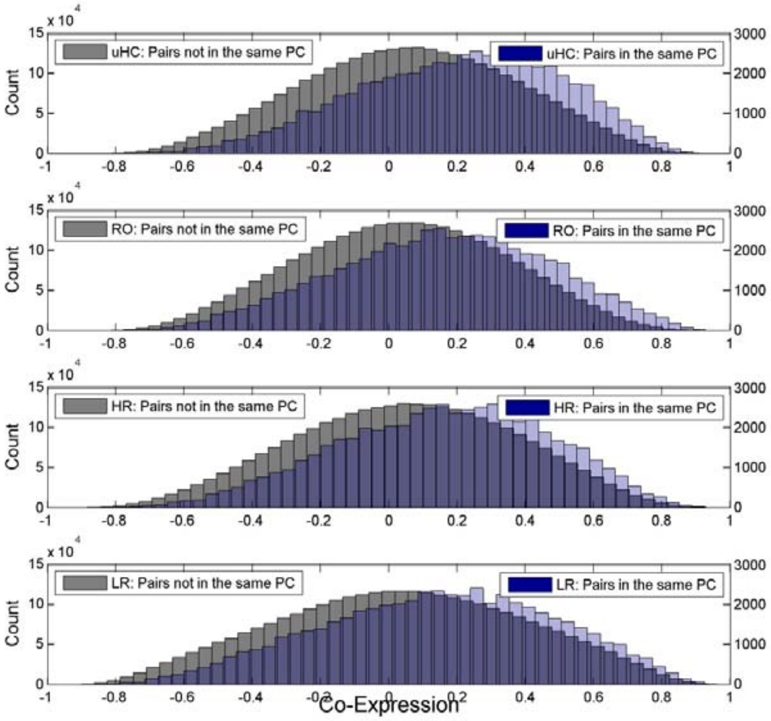
uHC: Pairs not in the same PC
uHC: Pairs in the same PC
RO: Pairs not in the same PC
RO: Pairs in the same PC
HR: Pairs not in the same PC
HR: Pairs in the same PC
LR: Pairs not in the same PC
LR: Pairs in the same PC
Count
x 10^4
Co-Expression

Figure S2

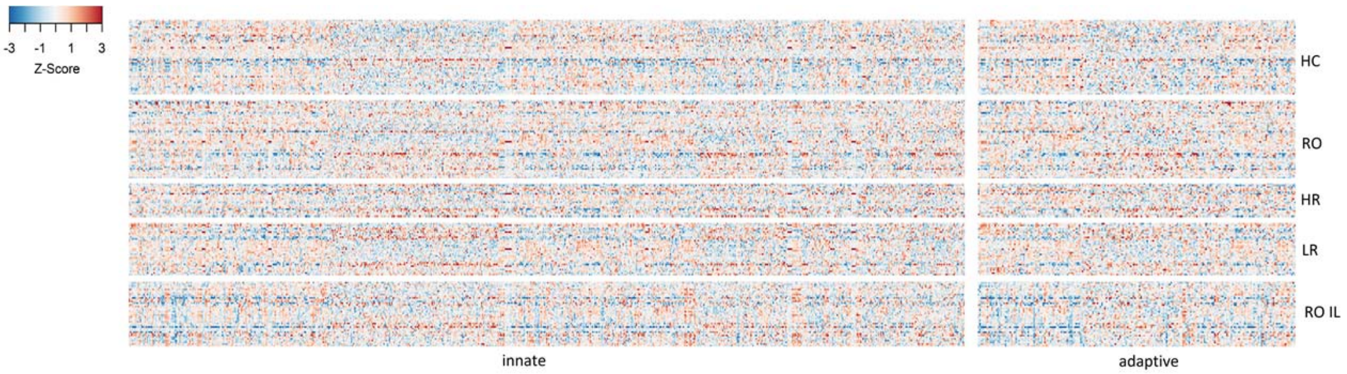

Figure S3

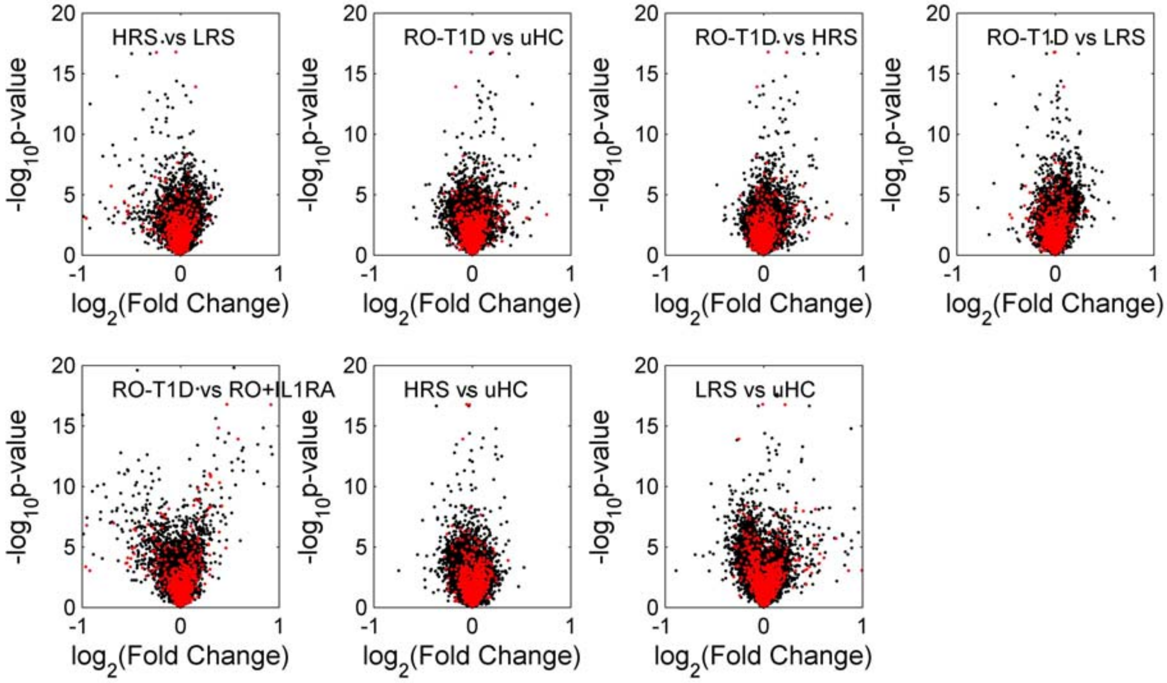
HRS vs LRS
RO-T1D vs uHC
RO-T1D vs HRS
RO-T1D vs LRS
RO-T1D vs RO+IL1RA
HRS vs uHC
LRS vs uHC
$-\log_{10}$p-value
$\log_2$(Fold Change)
0
5
10
15
20
-1
0
1

Figure S4

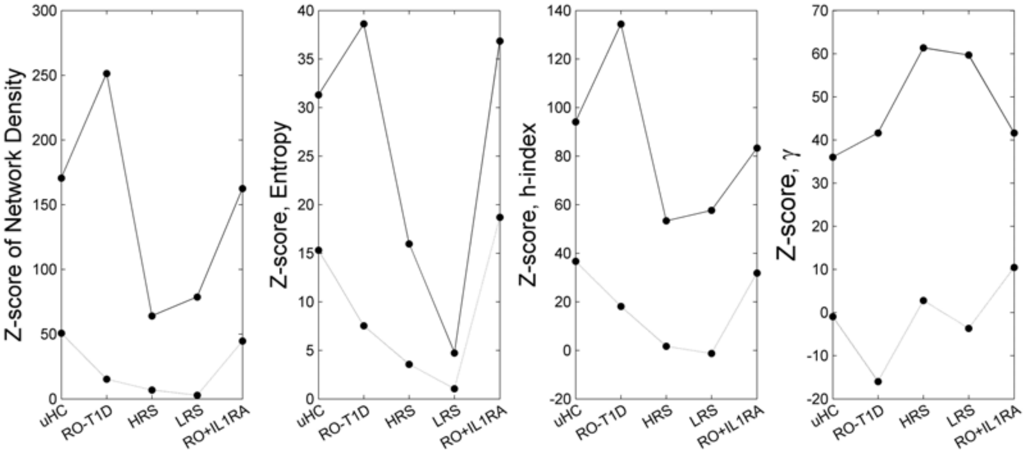
Z-score of Network Density
Z-score, Entropy
Z-score, h-index
Z-score, γ
uHC
RO-T1D
HRS
LRS
RO+IL1RA

Figure S5

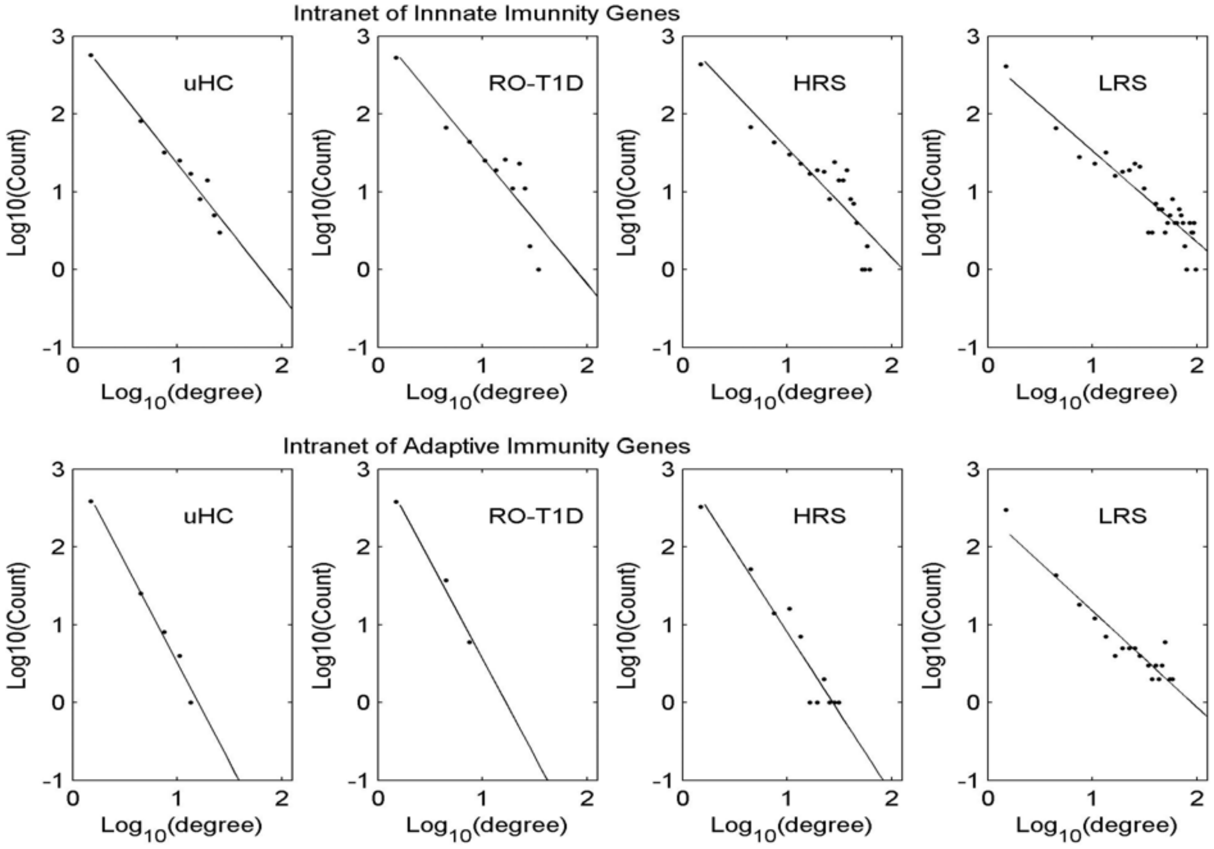
Intranet of Innnate Imunnity Genes
uHC
RO-T1D
HRS
LRS
Log10(Count)
Log10(degree)
Intranet of Adaptive Immunity Genes
uHC
RO-T1D
HRS
LRS
Log10(Count)
Log10(degree)
3
2
1
0
-1
0
1
2

Figure S6

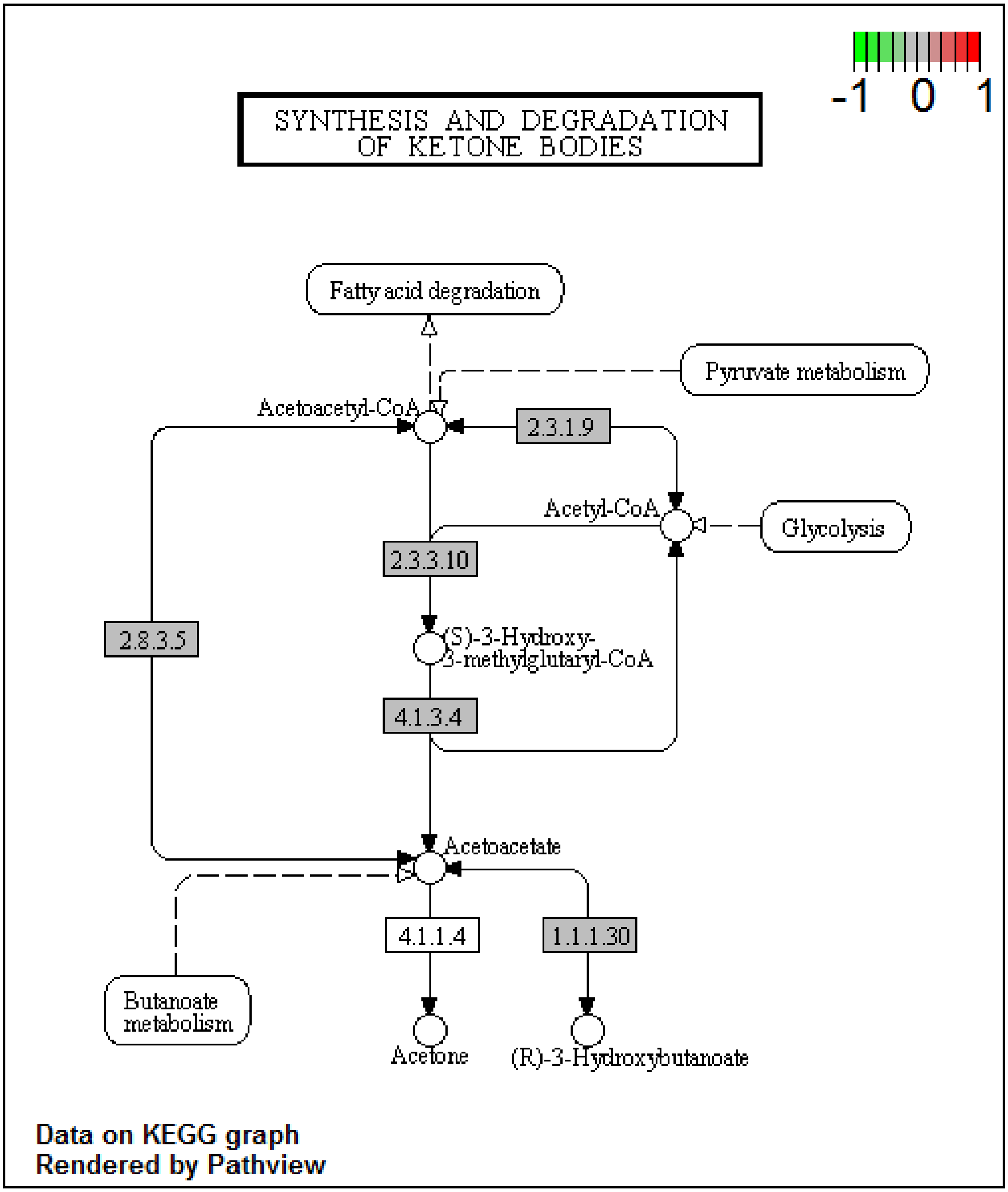
-1 0 1
SYNTHESIS AND DEGRADATION OF KETONE BODIES
Fatty acid degradation
Pyruvate metabolism
Acetoacetyl-CoA
2.3.1.9
Acetyl-CoA
Glycolysis
2.3.3.10
2.8.3.5
(S)-3-Hydroxy-3-methylglutaryl-CoA
4.1.3.4
Acetoacetate
4.1.1.4
1.1.1.30
Butanoate metabolism
Acetone
(R)-3-Hydroxybutanoate
Data on KEGG graph
Rendered by Pathview

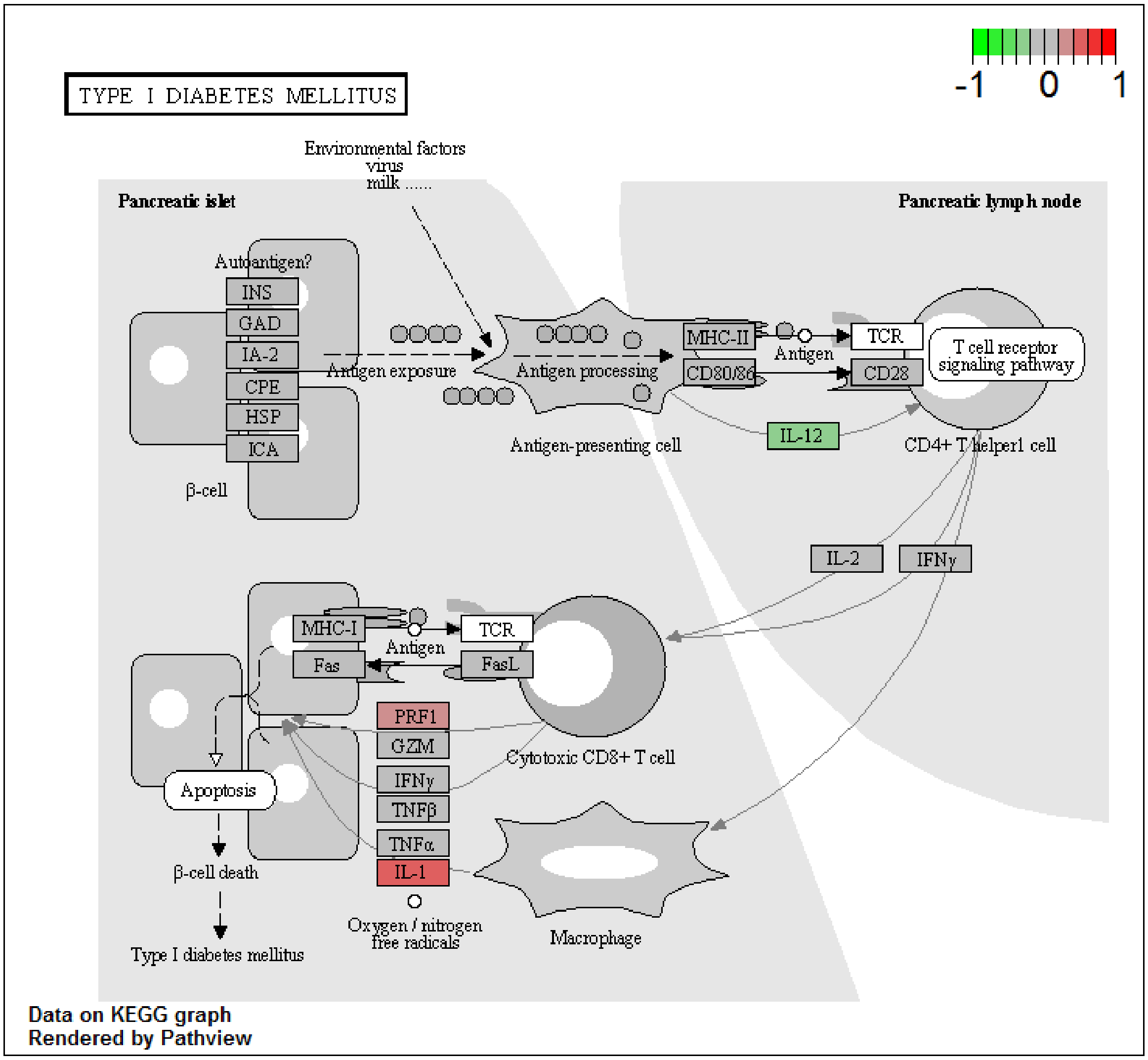
-1
0
1
TYPE I DIABETES MELLITUS
Environmental factors
virus
milk ......
Pancreatic islet
Pancreatic lymph node
Autoantigen?
INS
GAD
IA-2
CPE
HSP
ICA
β-cell
Antigen exposure
Antigen processing
Antigen-presenting cell
MHC-II
CD80/86
Antigen
TCR
CD28
T cell receptor signaling pathway
IL-12
CD4+ T helper1 cell
IL-2
IFNγ
MHC-I
Fas
Antigen
TCR
FasL
PRF1
GZM
IFNγ
TNFβ
TNFα
IL-1
Cytotoxic CD8+ T cell
Apoptosis
β-cell death
Type I diabetes mellitus
Oxygen / nitrogen free radicals
Macrophage
Data on KEGG graph
Rendered by Pathview

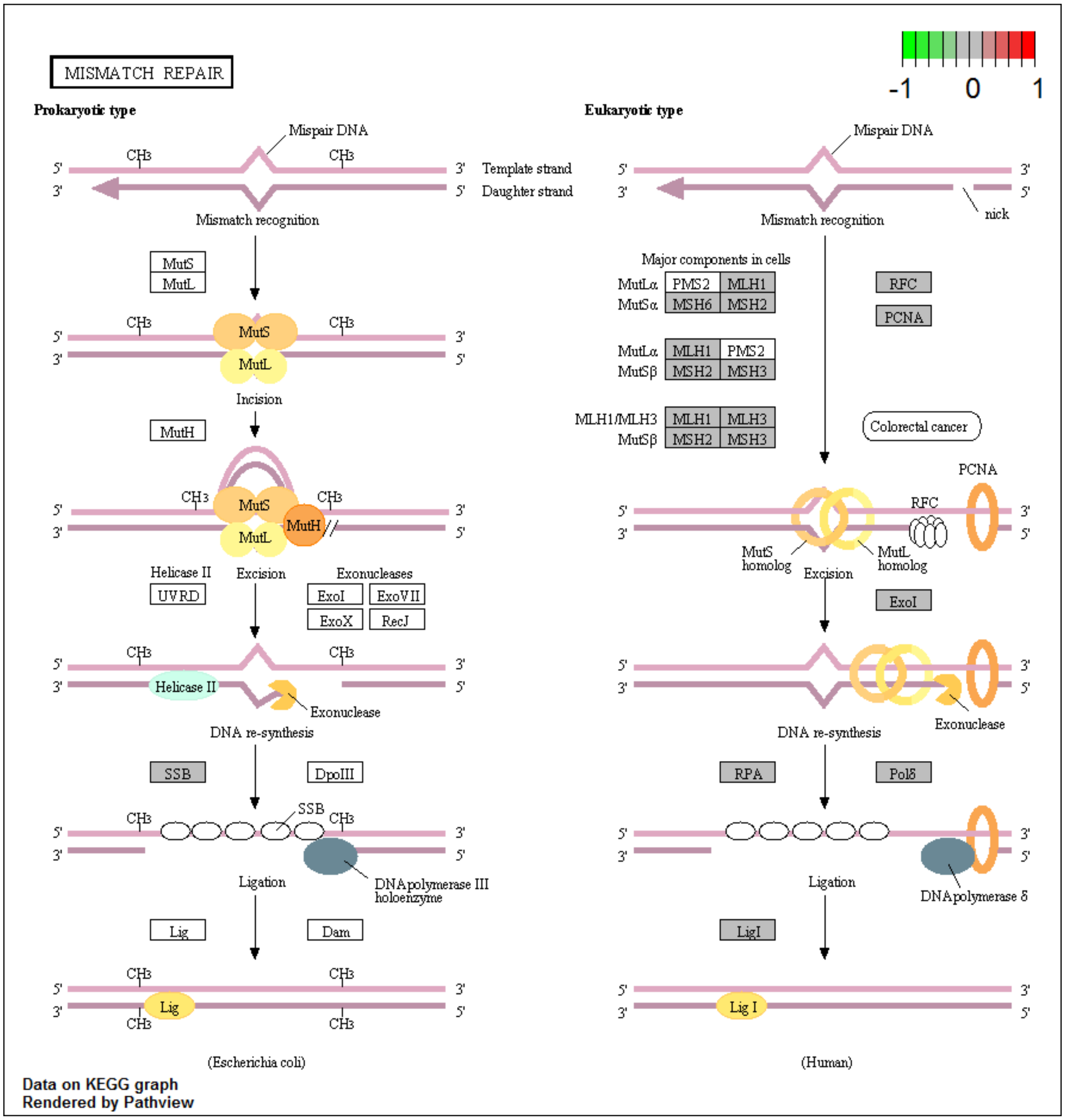
MISMATCH REPAIR
-1
0
1
Prokaryotic type
Eukaryotic type
Mispair DNA
CH3
5'
3'
Template strand
Daughter strand
nick
Mismatch recognition
MutS
MutL
Major components in cells
MutLα
PMS2
MLH1
MutSα
MSH6
MSH2
RFC
PCNA
MutLα
MLH1
PMS2
MutSβ
MSH2
MSH3
MLH1/MLH3
MLH1
MLH3
MutSβ
MSH2
MSH3
Colorectal cancer
Incision
MutH
PCNA
RFC
MutS homolog
MutL homolog
Helicase II
UVRD
Excision
Exonucleases
ExoI
ExoVII
ExoX
RecJ
ExoI
Helicase II
Exonuclease
DNA re-synthesis
SSB
DpoIII
RPA
Polδ
DNA polymerase III holoenzyme
DNA polymerase δ
Ligation
Lig
Dam
LigI
Lig I
(Escherichia coli)
(Human)
Data on KEGG graph
Rendered by Pathview
